# Cost-effectiveness of rhythm control strategy: Ablation versus antiarrhythmic drugs for treating atrial fibrillation in Korea based on real-world data

**DOI:** 10.3389/fcvm.2023.1062578

**Published:** 2023-01-24

**Authors:** Woojin Kim, Min Kim, Yun Tae Kim, Woongbi Park, Jin-bae Kim, Changsoo Kim, Boyoung Joung

**Affiliations:** ^1^Department of Preventive Medicine, Yonsei University College of Medicine, Seoul, Republic of Korea; ^2^Division of Cardiology, Department of Internal Medicine, Chungbuk National University Hospital, Cheongju, Republic of Korea; ^3^Department of Public Health, Yonsei University Graduate School, Seoul, Republic of Korea; ^4^Division of Cardiology, Department of Internal Medicine, Kyung Hee University Hospital, Seoul, Republic of Korea; ^5^Institute of Human Complexity and Systems Science, Yonsei University, Incheon, Republic of Korea; ^6^Division of Cardiology, Department of Internal Medicine, Severance Cardiovascular Hospital, Yonsei University College of Medicine, Seoul, Republic of Korea

**Keywords:** atrial fibrillation, rhythm control, cost-effectiveness, ablation, antiarrhythmic drugs

## Abstract

**Background:**

Ablation-based treatment has emerged as an alternative rhythm control strategy for symptomatic atrial fibrillation (AF). Recent studies have demonstrated the cost-effectiveness of ablation compared with medical therapy in various circumstances. We assessed the economic comparison between ablation and medical therapy based on a nationwide real-world population.

**Methods and findings:**

For 192,345 patients with new-onset AF (age ≥ 18 years) identified between August 2015 and July 2018 from the Korean Health Insurance Review and Assessment Service (HIRA) database, medical resource use data were collected to compare AF patients that underwent ablation (*N* = 2,131) and those administered antiarrhythmic drugs (*N* = 8,048). Subsequently, a Markov chain Monte Carlo model was built. The patients had at least one risk factor for stroke, and the base-case used a 20-year time horizon, discounting at 4.5% annually. Transition probabilities and costs were estimated using the present data, and utilities were derived from literature review. The costs were converted to US $ (2019). Sensitivity analyses were performed using probabilistic and deterministic methods. The net costs and quality-adjusted life years (QALY) for antiarrhythmic drugs and ablation treatments were $37,421 and 8.8 QALYs and $39,820 and 9.3 QALYs, respectively. Compared with antiarrhythmic drugs, incremental cost-effectiveness ratio of ablation was $4,739/QALY, which is lower than the willingness-to-pay (WTP) threshold of $32,000/QALY.

**Conclusion:**

In symptomatic AF patients with a stroke risk under the age of 75 years, ablation-based rhythm control is potentially a more economically attractive option compared with antiarrhythmic drug-based rhythm control in Korea.

## Introduction

With improvements in the rhythm control treatment of atrial fibrillation (AF), beneficial effects on cardiovascular (CV) outcomes have been shown in AF patients who received active rhythm control (1–3). Based on the latest available data from a national survey, the prevalence of AF in the Republic of Korea is increasing, and the proportion of patients at high risk for stroke or heart failure (HF) has been rising because of its prevalence in the aging society (4–6). The cost of disease burden is also increasing with AF-related complications in both in-hospital and outpatient clinic settings (4, 7).

Previous studies have revealed that ablation-based rhythm control of AF is associated with lower AF recurrences, prolonged time in sinus rhythm, and improved quality of life (8–11). In a trial of ablation vs. medical therapy in symptomatic patients with AF and HF, successful ablation could extend survival and reduce HF admission (12). Real-world studies have reported favorable outcomes, such as reduced ischemic stroke and death in ablated patients (13–16). It can be hypothesized that ablation-based rhythm control of AF is related to economic benefit. In some countries, economic assessments have reported a good cost-saving in ablation-based therapy compared with medical therapy (17, 18). However, the economic evaluation of different rhythm control strategies for treating AF has not been conducted in the Republic of Korea. Additional costs of drugs and ablation procedures, hospitalization, and reimbursement systems can impact economic assessment results (19). To provide acceptable economic value beyond clinical benefit (2, 3), we evaluated the cost-effectiveness of ablation-based and antiarrhythmic drugs (AADs)-based rhythm control therapies for patients with drug-refractory AF from a nationwide real-world cohort in the Republic of Korea.

## Materials and methods

This study is based on the National Health Insurance (NHI) claims database established by the Health Insurance Review and Assessment Service (HIRA) of the Republic of Korea (4–6, 20, 21). The NHI service (NHIS) is the single insurer controlled by the Korean government, and the majority (97.1%) of Korean population are its mandatory subscribers, with the remaining 3% of the population being medical aid subjects. The HIRA service is a value-based purchasing system for medical service quality improvement that provides a review of incurred medical costs and reports from healthcare providers about medical services performed for HIRA. These databases include the following: medical aid subjects, sociodemographic information of patients, their use of inpatient and outpatient services, procedure and procedure-related resource use, pharmacy dispensing claims, disease information, and mortality of the entire Korean population. This study was approved by the Institutional Review Board of the Kyung Hee University Health System (2020-05-040), and the requirement for informed consent was waived.

### Study population

In the NHIS data, 192,345 patients with newly diagnosed non-valvular AF were identified between 1 August 2015, and 31 July 2018. The data excluded those aged <18 or ≥75 years (*n* = 75,907) and with previous health events (*n* = 28,987) including HF, myocardial infarction (MI), ischemic stroke, intracranial hemorrhage (ICH), and gastrointestinal (GI) bleeding within 1-year of study enrollment. Subsequently, we set an inclusion criteria to mimic the CABANA trial (22) (*n* = 15,543) for the following reasons: (1) Prevalence of AF is rapidly increasing in people over 65 years of age, and (2) it is likely to be applicable to real-world patients we see in every practice. Additionally, patients who did not meet the indication of ablation therapy (*n* = 172) and who died or experienced the health events within 6 weeks after AF diagnosis (*n* = 9,382) were excluded. Eligible patients were divided into those who received ablation therapy (*n* = 2,131) and those who did not (*n* = 60,223). Among 60,223 patients, those without a history of rhythm control pharmacotherapy (*n* = 41,537) or having medication possession ratio (MPR) (23) of AADs less than 80% (*n* = 10,638) were excluded. Finally, the cost-effectiveness analysis included 2,132 and 8,048 patients treated with ablation-based and AADs-based rhythm control therapies, respectively (Supplementary Figure 1).

Atrial fibrillation was diagnosed using the International Classification of Diseases, 10*^th^* Revision (ICD-10) codes. Moreover, patients were designated to have AF only when it was a discharge diagnosis or confirmed more than twice in the outpatient department to ensure diagnostic accuracy (24). The AF diagnosis has previously been validated in the NHIS database with a positive predictive value of 94.1% (4, 25–28). Detailed definitions of AF, health events, periprocedural complications, and AADs for rhythm control are presented in Supplementary Tables 1, 2.

### Decision model

**FIGURE 1:**
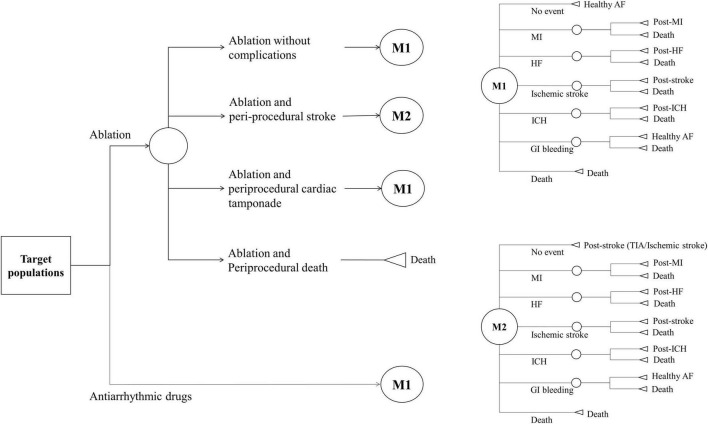
Markov model for decision analysis.

We developed a Markov chain Monte Carlo model to evaluate the cost-effectiveness of ablation-based and AADs-based rhythm control therapies in patients with newly diagnosed AF who were eligible for AF ablation. The analysis was conducted from the perspective of a healthcare provider. Data sources, except health utility, were obtained from the present study data, which reflect real-world practice. Because the time horizon was set to 20 years in the base case modeling, patients aged ≥ 75 years were excluded to reflect the post-procedure lifetime cost-effectiveness. The modeled health events included healthy AF, hospitalization or unplanned visits for HF, MI, ischemic stroke, ICH, GI bleeding, and death. We defined the fatality rate of each health event that transitioned to death. Patients who had experienced non-fatal health events transitioned to post-health event status with utility decrement, except for GI bleeding, which was assumed to be transited to healthy AF (7, 29). We assumed that the healthy state is transited annually based on the probability of an independent health event occurring within 1 year. All patients could experience recurrent health events during study period. Supplementary Figure 2 provides an overview of the constructed three-state Markov model. Patients undergoing ablation-based therapy could experience periprocedural stroke, cardiac tamponade, death, or no complications. Transition probabilities were estimated based on data from the Korean NHIS study subjects (Table 1 and Figure 1). Beginning the treatment with a mean age of patients, the cohort accrued costs and quality-adjusted life years (QALY) depending on the health state they inhabited each year. Discounting rate of 4.5% annually was applied to both costs and QALYs, reflecting the annual inflation rate in Korea (30). All unit costs were adjusted to US dollars using the exchange rate of 2019 (1298.7 KRW = 1 US dollar). The distribution of the parameters was assigned depending on the type of parameter.

**TABLE 1:** Model parameters.

Input	Base-case value (raw)	Base-case value (%/year)	Distribution in PSA	Reference
**Transition probabilities, ablation group**				
Major procedural complication	127/2,131	5.96	Normal	HIRA
TIA/ischemic stroke	88/2,131	4.13	Normal	HIRA
Cardiac tamponade	37/2,131	1.74	Normal	HIRA
Death within 30 days	2/2,131	0.09	Normal	HIRA
Events after procedure, first year*				
Hospitalization/unplanned visit for HF	638/2,129	29.97	Normal	HIRA
Myocardial infarction	28/2,129	1.32	Normal	HIRA
Ischemic stroke	71/2,129	3.33	Normal	HIRA
Intracranial hemorrhage	13/2,129	0.61	Normal	HIRA
Gastrointestinal bleeding	86/2,129	4.04	Normal	HIRA
All-cause mortality	11/2,129	0.52	Beta	HIRA
**Transition probabilities, AADs group**				
Events after AADs, first year*				
Hospitalization/unplanned visit for HF	1,320/8,048	16.40	Normal	HIRA
Myocardial infarction	74/8,048	0.92	Normal	HIRA
Ischemic stroke	163/8,048	2.03	Normal	HIRA
Intracranial hemorrhage	42/8,048	0.52	Normal	HIRA
Gastrointestinal bleeding	237/8,048	2.94	Normal	HIRA
All-cause mortality	101/8,048	1.25	Beta	HIRA
**Fatality**				
Hospitalization/unplanned visit for HF	50/1,958	2.55	Beta	HIRA
Myocardial infarction	8/102	7.84	Beta	HIRA
Ischemic stroke	16/234	6.84	Beta	HIRA
Procedure-related stroke	3/88	3.41	Beta	HIRA
Intracranial hemorrhage	14/55	25.45	Beta	HIRA
Gastrointestinal bleeding	12/323	3.72	Beta	HIRA
**Utility (QALYs)**				
Event-free AF	0.95 (0.93–0.98)		Beta	Reference
Hospitalization/unplanned visit for HF	0.73 (0.54–0.91)		Beta	Reference
Myocardial infarction	0.73 (0.58–0.88)		Beta	Reference
Ischemic stroke	0.60 (0.54–0.65)		Beta	Reference
Intracranial hemorrhage	0.67 (0.54–0.80)		Beta	Reference
**Cost (US $)**				
**Ablation-related (per event)**				
No complications	1,879.4		Gamma	HIRA
TIA/ischemic stroke	6,187.9		Gamma	HIRA
Cardiac tamponade	5,189.8		Gamma	HIRA
**Events related, ablation group (per year)**				
Hospitalization/unplanned visit for HF	3,217.5		Gamma	HIRA
Myocardial infarction	6,545.8		Gamma	HIRA
Ischemic stroke	7,643.9		Gamma	HIRA
Intracranial hemorrhage	21,238.3		Gamma	HIRA
Gastrointestinal bleeding	3,739.9		Gamma	HIRA
**Events related, AADs group (per year)**				
Hospitalization/unplanned visit for HF	3,556.4		Gamma	HIRA
Myocardial infarction	5,955.6		Gamma	HIRA
Ischemic stroke	7,991.3		Gamma	HIRA
Intracranial hemorrhage	9,398.1		Gamma	HIRA
Gastrointestinal bleeding	4,418.0		Gamma	HIRA
**Annual cost after periprocedural TIA/ischemic stroke**				
Ablation group	1774.8			
**Annual cost of event-free AF**				
Ablation group	1016.8		Gamma	HIRA
AADs group	1221.5		Gamma	HIRA

AAD, antiarrhythmic drug; AF, atrial fibrillation; HF, heart failure; HIRA, Health Insurance Review and Assessment Service; PSA, probability sensitivity analysis; QALY, quality-adjusted life-year; TIA, transient ischemic attack.

*Difference in probability of occurrence of healthy events (in first year) between ablation group and the AADs group by Pearson’s chi-square test–Hospitalization/unplanned visit for HF, <0.0001; MI, 0.103; ischemic stroke, 0.0003; intracranial hemorrhage, 0.619; gastrointestinal bleeding, 0.010; all-cause mortality, 0.004.

### Costs and utilities

The costs were obtained from the publicly available HIRA service of the Republic of Korea. The economic analysis considered all direct medical costs for the therapies (single-event, medication, and maintenance costs). Indirect societal costs were not considered in this study. To measure the quality-adjusted survival, QALY was calculated by multiplying life years by utility scores derived from medical literature (31–34). By definition, death (resulting from any cause) had a QALY of 0, and the utilities of event-free AF, hospitalization for HF, MI, ischemic stroke, and ICH were 0.95, 0.73, 0.73, 0.60, and 0.67, respectively. We assumed that the QALY of non-fatal GI bleeding was equal to that of the event-free AF. Table 1 lists the costs and utilities.

### Cost-effectiveness analysis

An individual-level simulation analysis was conducted, and for each AF rhythm control strategy, the estimated net costs and QALYs were quantified over a period of 20 years. Incremental cost-effectiveness ratios (ICERs) were calculated by dividing the incremental costs by the incremental effectiveness as the ablation minus drug therapy difference in the mean lifetime. The willingness-to-pay (WTP) threshold was determined to be US $32,000/QALY to reflect gross domestic product per capita of South Korea ($31,617 in 2017, $33,423 in 2018, and $31,846 in 2019).

### Sensitivity and subgroup analysis

In this study, deterministic and probabilistic sensitivity analyses (PSA) were performed to evaluate the uncertainty of the model owing to the limitations of the available data. To explore the effect of the change in assumed input parameters during the study period, deterministic sensitivity analyses were performed using different parameter values. First, we evaluated the case where both cost and result were not discounted (discount rate of 0%) and the case where discount rates of 3 and 7% were applied. In addition, a one-way sensitivity analysis was performed on all the parameters used in the model to identify inherent uncertainties and report their influence on the final ICER. The parameter values range one by one with 95% confidence intervals of utility and cost or ± 10% of the median value of transition probability. The discount rate applied ranged from 0 to 7%. The tornado diagram represents the impact on the ICER when varying a single parameter. The model parameters and assumptions for the distributions in PSA are shown in Supplementary Table 3. The simulation was run with Monte Carlo sampling for 10,000 replicates to develop parameters and a cost-effectiveness acceptability curve, assuming a WTP threshold. All analyses were performed in TreeAge Pro Healthcare 2022 R1.2 (TreeAge Software, Inc., Williamstown, MA, USA) and SAS 9.4 (SAS Institute Inc., Cary, NC, USA).

## Results

### Patient-level data

During the study period, 10,179 patients with AF receiving rhythm control therapy were simulated for over 20 years. The patients had a mean age of 62 years, 57% of them were male, and 64% had a CHA_2_DS_2_-VASc score ≥ 2, which implies a high risk of stroke. Complications related to ablation included periprocedural stroke, cardiac tamponade, and death within 30 days of the procedure.

### Base-case analysis

Table 1 present the base-case values of transition probability, fatality, utility, and cost. The rate of periprocedural complication was 5.96%/year, and periprocedural stroke was identified with the highest rate of 4.13%/year. Two periprocedural deaths were identified in the ablation group. During the first year after rhythm control treatments, there were higher rates of hospitalization/unplanned visits for HF (29.97 vs. 16.4%), MI (1.32 vs. 0.92%), ischemic stroke (3.33 vs. 2.03%), ICH (0.61 vs. 0.52%), GI bleeding (4.04 vs. 2.94%), and lower rate of all-cause mortality (0.52 vs. 1.25%) in the ablation group compared with the AADs group. The fatality was high in the order of ICH (25.45%), MI (7.84%), ischemic stroke (6.84%), GI bleeding (3.72%), periprocedural stroke (3.41%), and hospitalization/unplanned visits for HF (2.55%), and the overall fatality was 3.73%.

During a time horizon of 20 years, the total cost for a patient treated with ablation was $39,820 and that for a patient treated with antiarrhythmic drugs was $37,421; the greater costs of the ablation strategy were driven by the costs attributable to ICH and procedure-related complications. The average lifetime QALY for a patient treated with ablation was 9.3 and that for a patient treated with AADs was 8.8 (Table 2).

**TABLE 2:** Base-case and probabilistic sensitivity analyses (PSA) result of AF rhythm control strategies.

Treatment strategy	Number treated	Total costs (US $)	Total QALYs	ICER (US $/QALYs)
**Base-case analysis**				
AADs	8,048	37,421	8.8	Reference
Ablation	2,131	39,820	9.3	4738.51
**Probabilistic sensitivity analysis**				
AADs	8,048	37,316 *(8,803 to 126,640)	8.8 *(7.5 to 9.9)	Reference
Ablation	2,131	39,892 *(31,259 to 50,856)	9.3 *(7.7 to 10.6)	3,873.65 *(938.75 to 6808.55)

AAD, antiarrhythmic drug; AF, atrial fibrillation; ICER, increment cost-effectiveness ratio; QALY, quality-adjusted life-year.

*Uncertainty interval was estimated in probabilistic sensitivity analysis.

**FIGURE 2:**
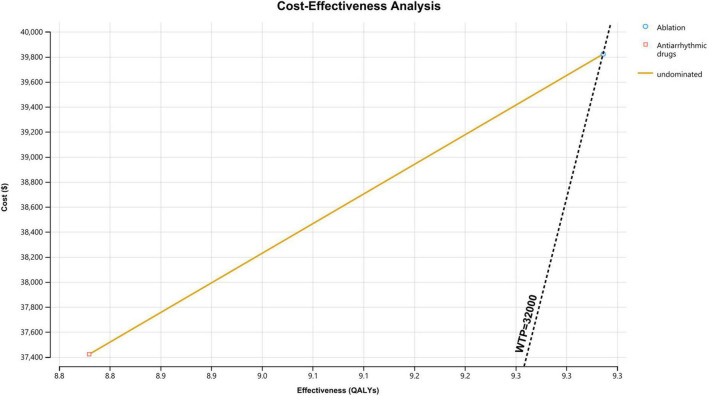
Cost-effectiveness analysis curve of ablation vs. antiarrhythmic drugs for treating atrial fibrillation. WTP, willingness to pay; QALY, quality-adjusted life year.

In the base-case assumptions, the ablation strategy showed better health outcomes (with a difference of 0.5 QALYs) and higher costs (with a difference of $2,399) than the AADs group. The ICER for ablation-based strategy was $4,739/QALY, which is below the assumed WTP threshold ($32,000/QALY); this result indicates that the ablation treatment is more cost-effective compared with antiarrhythmic drug therapy (Figure 2).

### Sensitivity analysis

**FIGURE 3:**
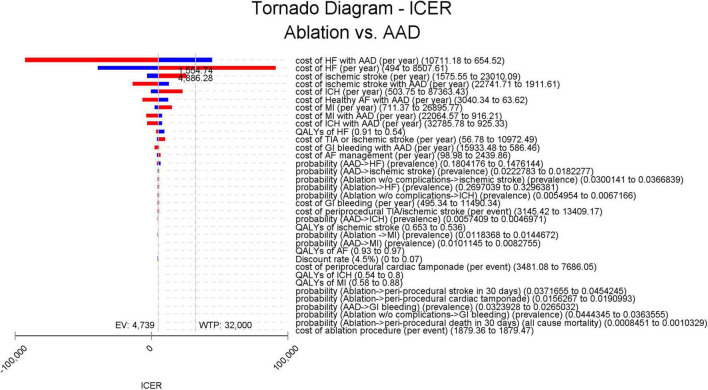
Tornado diagram for deterministic sensitivity analysis. The vertical line represents the ICER in the base-case analysis (US $4,738.5/QALY) and WTP thresholds (US $32,000/QALY), respectively, and the horizontal bars represent the variation of the ICER given the variations in parameters driving the model outcomes. The ranges of the variations are represented by the lower (blue bars) and higher (red bars) ICER values. AAD, antiarrhythmic drug; AF, atrial fibrillation; EV, expected value; GI, gastrointestinal; HF, heart failure; ICER, incremental cost-effectiveness ratio; ICH, intracranial hemorrhage; MI, myocardial infarction; QALY, quality-adjusted life-year; TIA, transient ischemic attack; WTP, willingness to pay.

In the 1-way sensitivity analysis based on differences in discount rates, ablation remained cost-effective over all tested discount rates (0, 3, and 7%), with an ICER ranging from $4,530/QALY to $4,751/QALY (Supplementary Table 4). The relative importance of each parameter is illustrated in a tornado diagram. We observed that the cost of hospitalization/unplanned visits HF was the critical variable with the greatest ICER range. However, the model did not appear to be sensitive to the other parameters (Figure 3).

**FIGURE 4:**
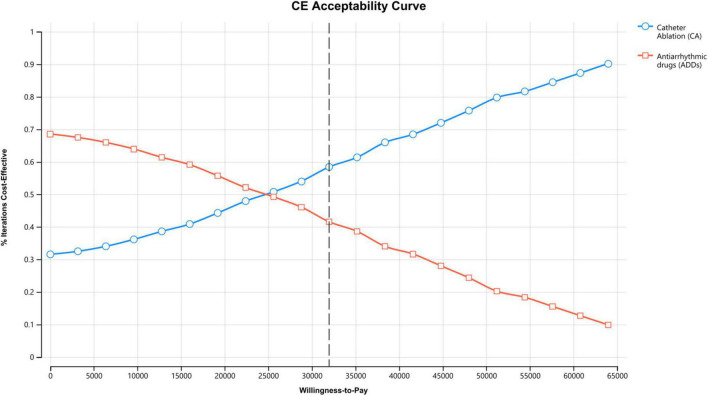
Cost-effectiveness acceptability curve of ablation vs. antiarrhythmic drugs for treating atrial fibrillation (AF).

PSA showed that the ICER was robust (Table 2). In the acceptability curves for ablation and AADs, the ICERs of ablation compared with AADs were below the WTP by 10,000 replications, indicating that ablation is cost-effective or cost-saving (Figure 4). The incremental cost-effectiveness bootstrap scatter plot shows plots of the incremental cost and effectiveness pairs for ablation vs. AADs. The points are distributed at 69.0% in the northeast and 30.9% in southeast quadrant of the cost-effectiveness plane, indicating that ablation has a higher total cost than AADs, but it is more effective with a 60% likelihood of meeting a $32,000/QALY WTP threshold (Supplementary Figures 3, 4).

## Discussion

The results of our analysis provide real-world population-based evidence confirming that choosing ablation-based, rather than AADs-based, rhythm control therapy in Korean patients with AF produces extra QALYs at a cost that meets current criteria for a good value in healthcare. Deterministic and PSA also revealed that ablation-based therapy was more cost-effective when using different parameter values, except for the costs of hospitalization/unplanned visits for HF and bootstrap uncertainty analysis. These findings support the economic benefit of ablation-based therapy along with an improvement in the clinical outcomes compared with AADs-based therapy.

Despite advances in ablation and procedure techniques, cost-effectiveness analyses of ablation-based rhythm control therapy have yielded heterogeneous conclusion that depend on the analysis model, parameters derived from the literature, and study population (17, 18, 35–39). Recently, Chew et al. (18) reported randomized trial-based economic evaluation results suggesting that catheter ablation of AF is economically attractive compared with drug therapy with an ICER of $57,893/QALY using the conventional WTP threshold of $100,000/QALY in the US. This study included detailed and comprehensive resource use data and quality-of-life adjustment factors. Patients assigned to the ablation group had significantly higher costs attributed to the initial cost of the procedure compared with the AADs group in the first 3-months of follow-up; no significant difference in costs was observed beyond 1 year suggesting the long-term economic benefits of ablation.

Leung et al. (17) reported the comparison of cost-effectiveness of catheter ablation and medical therapy using the National Health Service data in the United Kingdom. The authors concluded that their base case ICER was favorable for ablation-based therapy with a highly significant decrease in CV events and AF recurrences, despite a higher up-front cost for the procedure. These two studies also evaluated the cost-effectiveness of pre-specified subgroups of patients with HF. In both the studies, compared with the base case analysis, the value of ablation-based rhythm control was particularly attractive in the HF group based on greater quality-of-life gains.

With a view of negative cost-saving, Reynolds et al. (37) found that the cost-effectiveness of cryoballoon ablation, compared with AADs, has an ICER value greater than the WTP threshold in the UK. A study conducted in Australia by Gao et al. (36) reported the cost-effectiveness of catheter ablation vs. medical therapy in patients with AF and HF, yielding an ICER above the WTP threshold. However, these two study groups did not consider detailed CV outcomes, such as hospitalization for HF, stroke, and bleeding, which impact the costs and quality of life.

The periprocedural transient ischemic attack (TIA) or ischemic stroke rate was 4.13%/year in our population. This finding appears to be high compared to the majority of studies available from literature (22, 40, 41). The difference might have originated from definition of the periprocedural complications. We defined the periprocedural TIA or ischemic stroke based on hospital discharge diagnoses and the presence of brain imaging codes. The possibility of overestimation cannot be excluded because of the asymptomatic embolic events that showed a high incidence after AF ablation in previous study (42). Recent meta-analysis reported that the incidence of silent cerebral embolism after AF ablation ranged from 10 to 24%, respectively (43). These are considered potential reasons.

Our study evaluated the economic attractiveness of ablation-based and AADs-based rhythm control therapies for treating AF using comprehensive and detailed parameters in a real-world population. Our results support the greater cost-saving ability of ablation-based therapy compared with AADs-based therapy. Additionally, as shown in the previous studies and our study, ablation-based therapy is safe, with low rates of complications and mortality. These results are consistent with the outcomes of the sensitivity analyses.

## Study limitations

Several limitations should be acknowledged when interpreting the results of this study. First, because details of rhythm status were not available, we did not consider rhythm status in the model structure. However, we included patients in the antiarrhythmic drug therapy group who had an MPR ≥ 80%, assuming good rhythm control status. Second, repeat procedures and same day discharge after procedure were not considered in the model. In the clinical era, it is possible that patients may experience repeated ablations, and the cost of these procedures may vary from that of the index ablation. In Korea, it is uncommon to discharge same day after AF ablation. These could have resulted in the underestimation of the difference in costs between the two groups. Third, our results are derived from an on-treatment design that does not allow crossover between arms, which frequently occurs. We also analyzed specific subset of population who had no previous health events and <75 years. Therefore, this study may not accurately reflect situations commonly encountered in clinical practice. Fourth, we analyzed the health events of hospitalization/unplanned visits for HF, MI, ischemic stroke, ICH, GI bleeding, and mortality over 3 years; however, this duration does not fully reflect the rest of the patient’s life. Fifth, the utility of periprocedural TIA was equal to that of periprocedural strokes; however, the actual utility of periprocedural TIA may be better than that of the periprocedural strokes. Therefore, the QALY value of the ablation group may have been underestimated. Sixth, the model parameters and clinical event rates were derived from a country-based population and reimbursement system. From a generalizability perspective, our findings may not be applicable to a global region. Finally, the ablation-based rhythm control therapy included different ablation techniques, i.e., cryoballoon ablation and radiofrequency catheter ablation, and the cost-effectiveness of the different ablation techniques is indistinguishable in the analyses. Owing to a low number of cryoballoon ablations (3.43%), additional analyses were not considered. The different costs of the different tools, such as radiofrequency with different mapping systems and ablation/mapping catheters, cryoballoon, could potentially grow the costs of ablation and will become even more important with upcoming pulsed-field ablation therapies.

## Conclusion

Based on the real-world economic evaluation, ablation-based rhythm control therapy for treating AF under the age of 75 years is a cost-effective therapeutic option compared with the antiarrhythmic drugs-based therapy, with an incremental cost-effectiveness ratio of $7,913/QALY. This result was consistent regardless of the stroke risk and presence of HF.

## Data availability statement

The raw data supporting the conclusions of this article will be made available by the authors, without undue reservation.

## Ethics statement

This study was approved by the Institutional Review Board of the Kyung Hee University Health System (2020-05-040), and the requirement for informed consent was waived. The ethics committee waived the requirement of written informed consent for participation.

## Author contributions

BJ, MK, and WK: conceptualization and writing—original draft. YK: data curation. WK and YK: formal analysis. BJ and J-BK: funding acquisition and project administration. BJ, MK, WK, and YK: investigation. WK, MK, J-BK, CK, and BJ: methodology. WK, YK, and WP: resources. CK, BJ, MK, WK, and WP: software. CK and BJ: supervision. CK, BJ, MK, and WK: validation, visualization, and writing—review and editing. All authors contributed to the article and approved the submitted version.
