# Supplementary material for: Cost-effectiveness of rhythm control strategy: Ablation versus antiarrhythmic drugs for treating atrial fibrillation in Korea based on real-world data
Source: Front Cardiovasc Med. 2023 Jan 24;10:1062578. doi: 10.3389/fcvm.2023.1062578 (PMC9902500; doi:10.3389/fcvm.2023.1062578)
Supplement: Supplementary file 1 [file Data_Sheet_1.PDF]

# Supplemental materials

**Supplementary Table 1. Operational definition of events**

| Outcomes | ICD-10 codes | Claim codes | Disease /Procedure | Inclusion criteria |
|---|---|---|---|---|
| **Periprocedural complications (in ablation group)** | | | | |
| TIA/ischemic stroke | I63 | - CT brain: HA451, HA461, HA471, HA481<br>- MRI brain: HE101, HE201, HE401, HE501<br>- MR angiography: HE135, HE235, HE535 | Cerebral infarction or claims for brain imaging (CT, MRI) | During hospitalization or within 30 days of procedure |
| | I64 | | | |
| | G45, G45.8, G45.9 | | TIA or claims for brain imaging (CT, MRI) | |
| Cardiac tamponade | | C8060, C8061 | Claims for pericardiocentesis | During hospitalization |
| Death | | | All cause death | Within 30 days of procedure |
| **Health events** | | | | |
| Hospitalization/unpanned visit for HF | I50 | | Heart failure | Admission≥1 or outpatient department≥2 |
| Myocardial infarction | I21 | | Acute myocardial infarction | Admission≥1 or outpatient department≥2 |
| | I22 | | Subsequent myocardial infarction | |
| Ischemic stroke | I63 | | Cerebral infarction | Admission≥1 or outpatient department≥2 + brain imaging (CT, MRI) |
| | I64 | | | |
| Intracranial hemorrhage | I60 | | Subarachnoid hemorrhage | Admission≥1 or outpatient department≥2 + brain imaging (CT, MRI) |
| | I61 | | Intracerebral hemorrhage | |

| | | | | |
|---|---|---|---|---|
| | I62 | | Other nontraumatic intracranial hemorrhage | |
| Gastrointestinal bleeding | K25 (subcodes 0,2,6 only) | | Gastric ulcer | Admission≥1 or outpatient department≥2 |
| | K26 (subcodes 0,2,6 only) | | Duodenal ulcer | |
| | K27 (subcodes 0,2,6 only) | | Peptic ulcer, site unspecified | |
| | K28 (subcodes 0,2,6 only) | | Gastrojejunal ulcer | |
| | K62.5 | | Hemorrhage of anus and rectum | |
| | K92.0, K92.1, K92.2 | | Hematemesis, melena and unspecified GI bleeding | |
| | I85.0 | | Esophageal varices with bleeding | |
| | I98.3 | | Esophageal varices with bleeding in diseases classified elsewhere | |

*Abbreviations*: CT, computed tomography; GI, gastrointestinal; HF, heart failure; ICD-10, the International Classification of Disease 10th Revision; MRI, magnetic resonance imaging; TIA, transient ischemic attack.

**Supplementary Table 2. Generic name and ATC/HIRA code for antiarrhythmic drugs**

| Generic name | Brand name | Dose | ATC codes | HIRA codes |
|---|---|---|---|---|
| Flecainide | Tambocor Tab. | 50mg | C01BC04 | 159302ATB |
| | Fulcard Tab. | 50mg | | |
| Propafenone | Rytmonorm SR Cap. | 225mg | C01BC03 | 219503ACR |
| | Rytmonorm SR Cap. | 325mg | | 219504ACR |
| | Rytmonorm SR Cap. | 425mg | | 219504ACR |
| | Profenon Tab. | 150mg | C01BC03 | 219501ATB |
| | Profenon Tab. | 300mg | | 219502ATB |
| Pilsicainide | Sunrythm Cap. | 25mg | C01BG | 502101ACH |
| | Sunrythm Cap. | 50mg | | 502102ACH |
| Sotalol | Sotaron Tab. | 40mg | C07AA07 | 230402ATB |
| Dronedarone | Multaq Tab. | 426mg | C01BD07 | 597401ATB |
| Amiodarone | Codarone Tab. | 200mg | C01BD01 | 107401ATB |

*Abbreviations*: ATC, anatomical therapeutic chemical; Cap, capsule; HIRA, Health Insurance Review and Assessment Service; SR Cap., sustained release capsule; Tab., tablet.

**Supplementary Table 3. Information on parameters for probabilistic sensitivity analysis**

| Parameters | | Mean | SD |
|---|---|---|---|
| **Utility (QALY)** | Healthy AF | 0.95 | 0.01 |
| | Hospitalization/unpanned visit for HF | 0.73 | 0.09 |
| | Myocardial infarction | 0.73 | 0.08 |
| | Ischemic stroke | 0.60 | 0.03 |
| | Intracranial hemorrhage | 0.67 | 0.07 |
| **Ablation-related cost (per event)** | Catheter ablation without complications | 1879.41 | 0.03 |
| | Periprocedural TIA/ischemic stroke | 6187.85 | 5048.14 |
| | Periprocedural cardiac tamponade | 5189.80 | 1959.37 |
| **Event-related cost with ablation group (per year)** | Healthy AF | 3217.52 | 498.01 |
| | Hospitalization/unpanned visit for HF | 6545.83 | 9340.80 |
| | Myocardial infarction | 7643.90 | 1099.84 |
| | Ischemic stroke | 21238.33 | 2319.74 |
| | Intracranial hemorrhage | 3939.85 | 4469.53 |
| | GI Bleeding | 3217.52 | 498.01 |
| **Event-related cost with AADs group (per year)** | Healthy AF | 3556.36 | 4731.61 |
| | Hospitalization/unpanned visit for HF | 5955.55 | 6672.23 |
| | Myocardial infarction | 7991.34 | 974.38 |
| | Ischemic stroke | 9398.08 | 11677.44 |
| | Intracranial hemorrhage | 4418.00 | 4814.22 |
| | GI Bleeding | 3556.36 | 4731.61 |

*Abbreviations*: AAD, antiarrhythmic drug; AF, atrial fibrillation; GI, gastrointestinal; HF, heart failure; QALY, quality adjusted life year; SD, standard deviation.

**Supplementary Table 4. Results of 1-way sensitivity analysis assuming different discount rates**

| Alternatives | Cost ($) | Incremental Cost | Effectiveness (QALYs) | Incremental Effect | ICER | Category of dominance |
|---|---|---|---|---|---|---|
| **Discount rate = 0%** | | | | | | |
| AADs | 55,917 | Reference | 12.5 | Reference | Reference | Undominated |
| Ablation | 57,644 | 1,726 | 12.8 | 0.4 | 4,530.36 | Undominated |
| **Discount rate = 3%** | | | | | | |
| AADs | 42,402 | Reference | 9.8 | Reference | Reference | Undominated |
| Ablation | 44,639 | 2,237 | 10.3 | 0.5 | 4,750.76 | Undominated |
| **Discount rate = 7%** | | | | | | |
| AADs | 30,938 | Reference | 7.5 | Reference | Reference | Undominated |
| Ablation | 33,520 | 2,582 | 8.1 | 0.6 | 4,649.15 | Undominated |

*Abbreviations*: AAD, antiarrhythmic drug; ICER, incremental cost-effectiveness ratio; QALY, quality adjusted life year.

**Supplementary Figure 1. Flow chart**

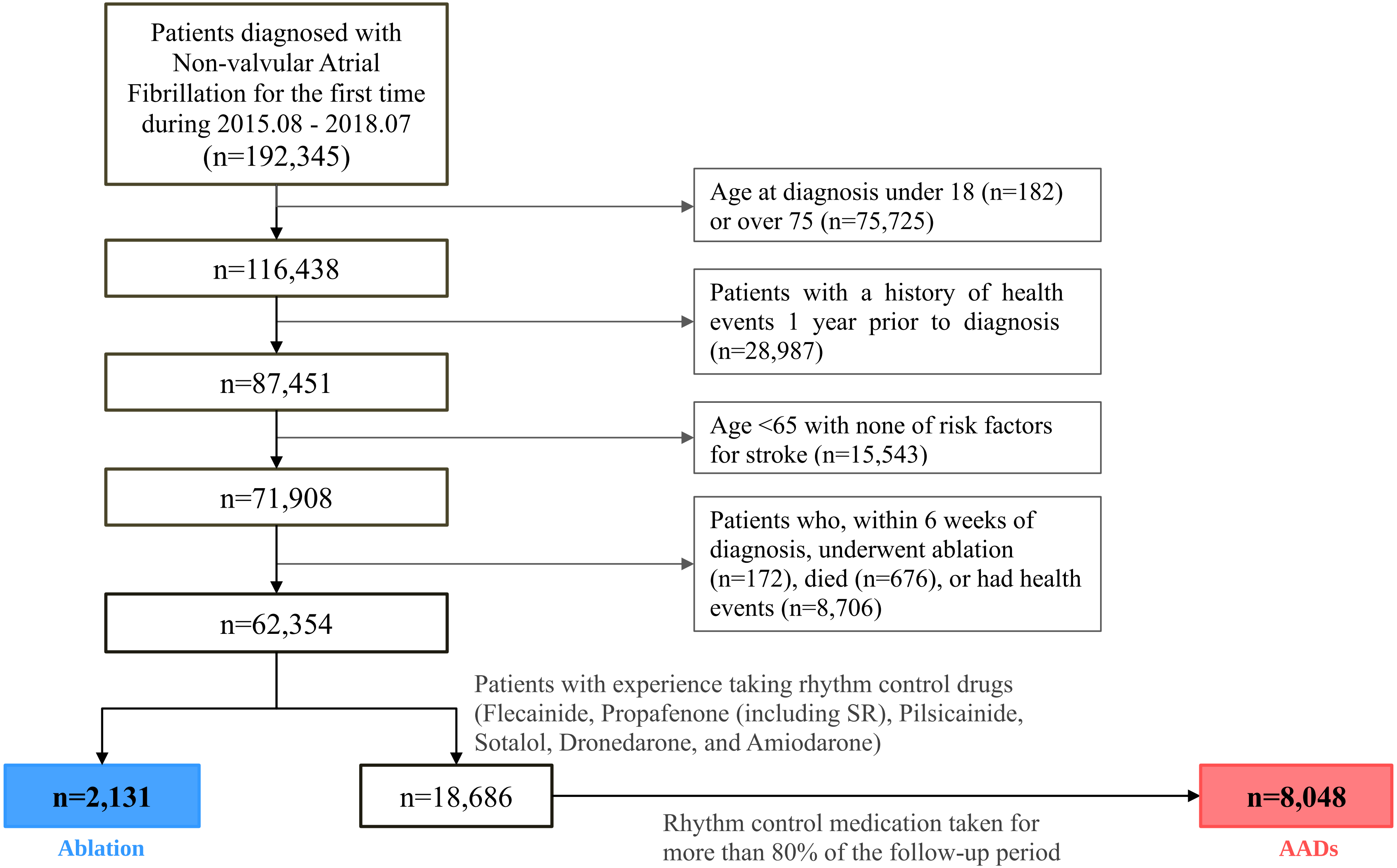


*Abbreviations*: AAD, antiarrhythmic drug; SR, sustained release.

**Supplementary Figure 2. Overview of three-state Markov model**

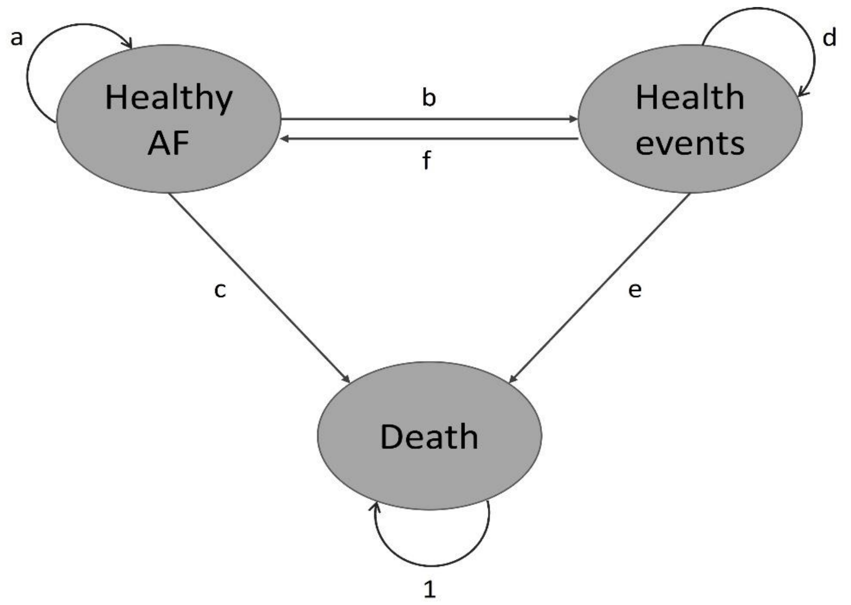


A three-state Markov model has been constructed. Based on the transition probability used in the analysis, when cohort simulation is performed on a yearly basis, the number of subjects in each state can be expressed by the following formula.

N: Number of baseline subjects (=$N_{W0}$)

$N_{W\ i}$ : Number of patients in well state (=healthy AF state) after i years

$N_{E\ i}$ : Number of patients in health event after i years

$N_{D\ i}$ : Number of patients dying after i years

(1) When the health event is HF, MI, IS, and ICH, a+b+c=1; d+e=1; and N=$N_0$.

① $a*N_{W(i-1)} = N_{W\ i}$

② $b*N_{W(i-1)} + d*N_{E(i-1)} = N_{E\ i}$

③ $c*N_{W(i-1)} + e*N_{E(i-1)} + N_{D(i-1)} = N_{D\ i}$

④ $N_{W\ i} + N_{E\ i} + N_{D\ i} = N$

(2) When the health event is GI bleeding, a+b+c=1; d+e+f=1; and N=$N_0$.

① $a*N_{W(i-1)} + f*N_{E(i-1)} = N_{W\ i}$

② $b*N_{W(i-1)} + d*N_{E(i-1)} = N_{E\ i}$

③ $c*N_{W(i-1)} + e*N_{E(i-1)} + N_{D(i-1)} = N_{D\ i}$

④ $N_{W\ i} + N_{E\ i} + N_{D\ i} = N$

**Supplementary Figure 3. Cost-effectiveness scatter plot of probabilistic sensitivity analysis**

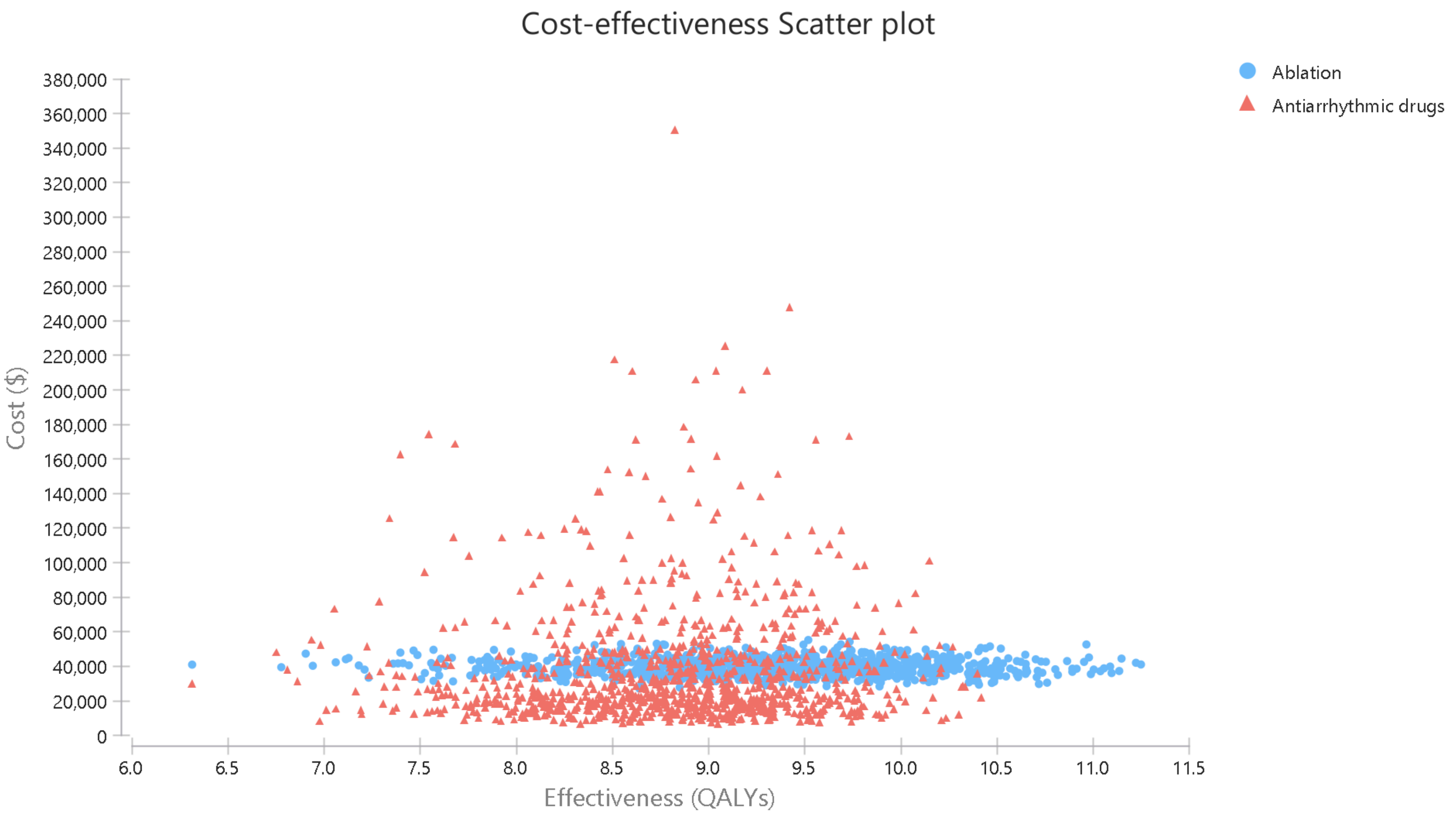

**Supplementary Figure 4. Incremental cost-effectiveness scatterplot**

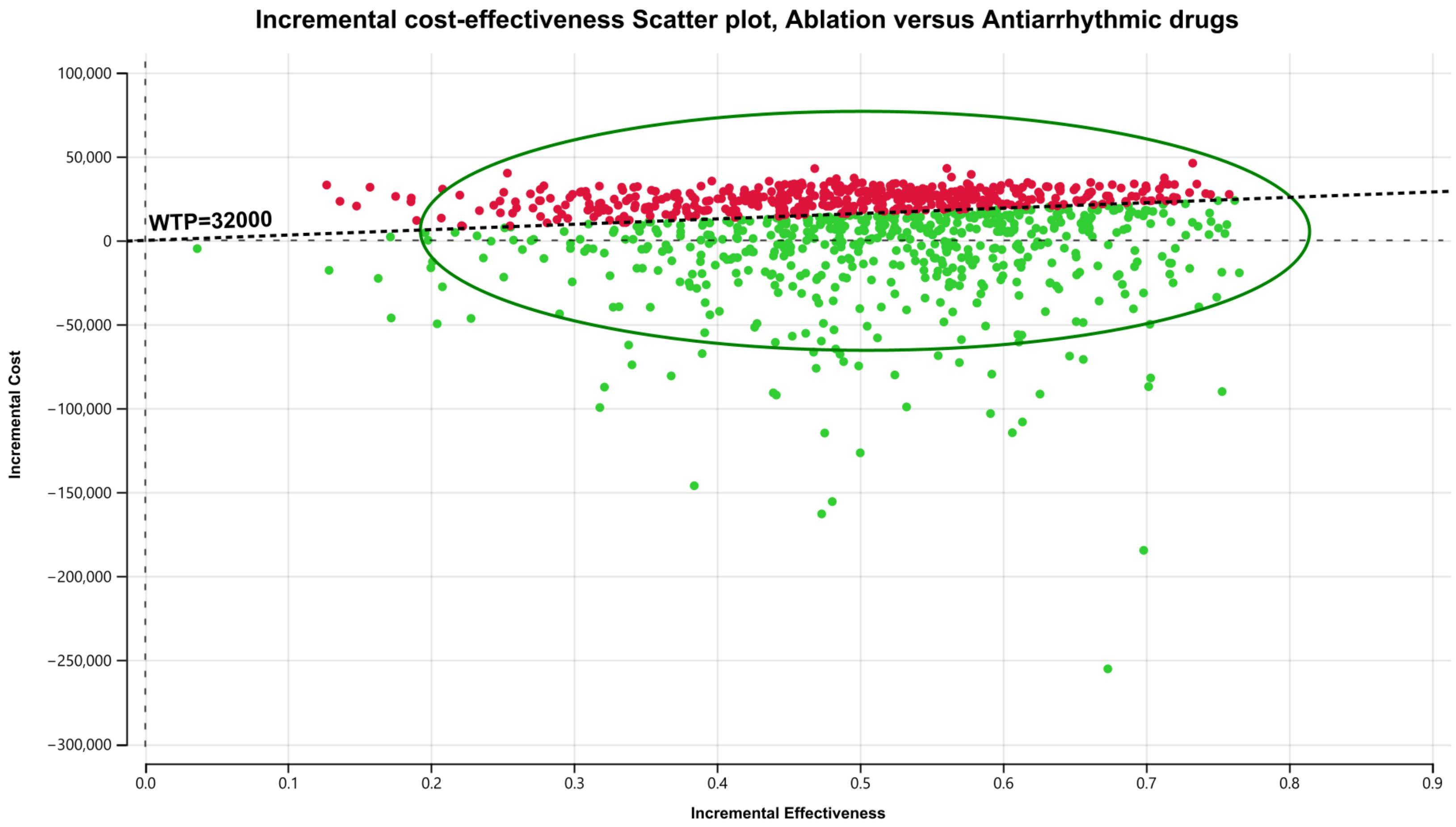


The dashed line denotes the threshold of $32,000 per QALY. The elliptical circles included 95% of simulations in the model. Green dots represent simulations below the $32,000 per QALY gained cost-effectiveness threshold while red dots represent simulations that are above this threshold and are not considered cost-effective
